# Inhibition of Endotoxin-Induced Hepatotoxicity by Melatonin in Rats

**Published:** 2008-06

**Authors:** Praveen Rishi, Sushma Bharrhan, Manmeet Pal Singh Bhalla, Ashwani Koul, Kanwaljit Chopra

**Affiliations:** 1 *Department of Microbiology, Panjab University, Chandigarh, India;*; 2 *Department of Biophysics, Panjab University, Chandigarh, India;*; 3 *University Institute of Pharmaceutical Sciences, Panjab University, Chandigarh, India*

**Keywords:** endotoxin, liver, melatonin, necrosis, oxidative stress, tumor necrosis factor α

## Abstract

Bacterial endotoxin or lipopolysaccharide causes extensive damage to various organs including the liver. This is due to an increased production of tumor necrosis factor α induced- reactive intermediates. These intermediates are known to cause extensive damage to a variety of cellular biomolecules leading to oxidative stress. In the present study, the role of the pineal hormone melatonin was evaluated as an antioxidant against endotoxin induced- hepatotoxicity using Wistar rats. Bacterial endotoxin was injected (i.v) and animals were sacrificed 8h post-challenge. Endotoxemia was associated with a statistically significant rise in the serum levels of alanine aminotransferase, aspartate aminotransferase, alkaline phosphatase and also caused histopathological changes. Administration of melatonin could significantly attenuate these enzymatic and associated histological alterations. Melatonin was administered (i.p) pre and/or post endotoxin challenge. A significant reduction in the levels of malondialdehyde and tumor necrosis factor-α in the hepatic tissue was also observed with melatonin supplementation. Reduction in the levels of endogenous antioxidants such as superoxide dismutase, catalase and reduced glutathione after endotoxin challenge was effectively attenuated by the administration of melatonin. Endotoxin challenge caused a marked increases in the levels of nitrite, and this was significantly lowered by melatonin administration. The above mentioned changes might have resulted in endotoxin associated hepatocellular necrosis which was minimized by melatonin supplementation in the present study.

## INTRODUCTION

Most of the toxic manifestations induced by Gram negative bacteria are due to their outer membrane component i.e. lipopolysaccharide (LPS), also known as endotoxin (1). Endotoxic shock is an important clinical entity characterized by hypotension and vascular damage which mediates intravascular coagulation leading to multiple organ failure resulting in death (2, 3).

Liver integrity and functions are crucial for survival of patients suffering from trauma, operations or infections (4). Since the liver is implicated in almost all biological processes, its damage can have severe impacts on metabolism, the immune response, detoxification processes, and antimicrobial defense. Despite rapid advances in the treatment of endotoxic shock, mortality caused by endotoxin remains high (5). It has also been reported that the present antibiotic therapy does not prevent the toxic manifestation and may even promote the release of LPS from bacteria (1, 6). LPS interacts with Kupffer cells leading to their activation, resulting in the onset of liver damage by release of cytotoxic agents including tumour necrosis factor α (TNF-α). TNF-α, a well known multifactorial cytokine secreted by the inflammatory cells. It is involved in numerous pathological states including liver dysfunction. TNF-α stimulates production of reactive oxygen species (ROS) and reactive nitrogen intermediates (RNIs) by activated macrophages causing liver damage due to the oxidative effects. Additionally, LPS induces migration of activated polymorphonuclear leukocytes (PMNs) into the liver which constitutes another source of free radicals. The oxidative stress thus generated, inactivates superoxide dismutase, catalase and glutathione peroxidase, enzymes that are involved in detoxification of toxic free radicals (7). Therefore, to combat the oxidative stress, scientific interest has been directed towards various antioxidants including melatonin.

Melatonin (N-acetyl-5-methoxytryptamine, melatonin) is the major product of the pineal gland, and plays a fundamental role in the neuroimmuno-endocrine system. Melatonin may also influence hematopoiesis, either by stimulating hemopoietic cytokines, including opioids, or by directly affecting specific progenitor cells, such as pre-B cells, monocytes, and natural killer cells. It has been proposed that melatonin may prophylactically stimulate the immune response, and strengthen immune reactivity (15). Melatonin participates in many important physiological functions, including anti-inflammation and immuno-regulation, as well as functioning as a broad spectrum antioxidant (8, 9). In addition, it also decreases free radical levels by stimulating the activities of enzymes involved in antioxidative defense (10, 11). Melatonin has been shown to protect the liver in several models of liver injury via inhibiting oxidative and nitrosative damage. Melatonin inhibits nitric oxide (NO) formation by inhibiting the activity of inducible NO synthase and hence prevents LPS-induced hepatotoxicity in endotoxemic rats (12, 13, 14). Protective effects of melatonin have been well documented against the symptoms of severe sepsis/shock in both animals and humans (15, 16). It has also been found to have protective role against immunological liver injury induced by *Bacillus Calmette-Guerin* plus lipopolysaccharide (9). Recently, it has been shown that melatonin protects against LPS-induced liver damage in galactosamine sensitized mice through its strong ROS-scavenging, anti-inflammatory and antiapoptotic effects (17). Therefore, in the present investigation, attempts were made to evaluate the effects of melatonin on endotoxin- mediated liver damage in a rat model in order to understand the underlying mechanism of amelioration of the disease.

## MATERIALS AND METHODS

### Agents

Lipopolysaccharide (LPS from *E. coli* serotype 0111:B4) and melatonin were obtained from Sigma Aldrich Chemicals, St. Louis, MO, USA. The preparations were made fresh every time before the commencement of the experiment.

### Establishment of hepatotoxicity in Wistar Rats

Female Wistar rats (150-200 g) were procured from Central Animal House, Panjab University, Chandigarh (India). The animals were allowed free access to food (Ashirwad Industries Pvt Ltd, India) and water *ad-libitum*. All experimental protocols were approved by the Institutional Animals Ethics Committee, Panjab University, Chandigarh (India).

### Experimental design to assess the effect of melatonin

Animals were divided into four main groups, each comprising of six rats. For all the groups, dose of LPS was prepared in sterile distilled water and that of melatonin was prepared in 0.25% ethanol in saline. (A) Control group: Rats in this group were administered normal saline i.p. (B) Melatonin *per se* group: Animals in this group received single i.p. dose of 10 mg melatonin/kg b. wt (dose standardized in our lab). (C) LPS challenged group: Animals in this group received a dose of LPS (10mg/kg b.wt) i.v. (D) LPS challenged and melatonin supplemented group: Rats in this group were further divided into three subgroups depending on the schedule of melatonin administration: i) LPS + MTb group: Each rat received a dose of melatonin 30 min before LPS challenge; ii) LPS + MTa group: Each rat received a dose of melatonin 2 h after LPS injection; iii) LPS + MT2 group: Animals in this group received two doses of melatonin. First dose of melatonin was given 30 min before, followed by second dose given 2 h after LPS challenge. Animals in all these groups were sacrificed at 8 h post-endotoxin challenge by cervical dislocation. Livers were removed quickly, washed in cold PBS and stored at -20°C till further use.

### Markers of liver damage

**Assessment of liver function.** Blood was collected by retro-orbital puncture from rats before they were sacrificed. Alanine aminotransferase (ALT) and aspartate aminotransferase (AST) were estimated by method recommended by International Federation of Clinical Chemistry (IFCC) using ERBA test kits (ERBA Diagnostics, Mannheim, Germany). Alkaline phosphatase (ALP) was estimated by the p-nitrophenyl phosphate method recommended by German Society for Clinical Chemistry using Enzopak Diagnostic kit (Reckon Diagnostics, India).

**Histopathological studies.** Liver tissues removed aseptically from all the groups were cut into small pieces and fixed in 10% buffered formalin. Samples were processed, stained with haematoxylin-eosin and examined under the light microscope.

**Extent of peroxidative damage.** The quantitative measurement of lipid peroxidation in liver was performed according to the method of Wills (18). The amount of malondialdehyde (MDA) formed which is a measure of lipid peroxidation, was assayed by the reaction with thiobarbituric acid (TBA). In brief, to 0.5 ml of liver homogenate, 0.5 ml of Tris-HCl buffer (0.1 M, pH7.4) was added and the mixture was incubated at 37°C for 2 h. Following incubation, 1.0 ml of 10% (w/v) ice-cold trichloroacetic acid (TCA) was added and the mixture was centrifuged at 1,000 rpm for 10 minutes. To 1.0 ml of supernatant (obtained after centrifugation), 1.0 ml of 0.67% (w/v) TBA was added and the mixture was kept in boiling water bath for 10 min. After cooling the tubes with tap water, 1.0 ml of distilled water was added and absorbance was measured at 532 nm. The results were expressed as nanomoles of MDA per milligram of protein, using the molar extinction coefficient of chromophore (1.56 × 10^5^ m^-1^cm^-1^) for which protein content of tissue homogenates was calculated according to the method of Lowry *et al*. (19).

**Estimation of reduced glutathione (GSH) levels.** GSH levels in the livers were estimated according to the method of Jollow *et al*. (20). 1.0 ml of liver homogenate was precipitated with 1.0 ml of 4% sulphosalicylic acid. The samples were kept at 4°C for at least 1 h, and then subjected to centrifugation at 1200 × g for 15 min at 4°C. The assay mixture contained 0.1 ml of supernatant, 0.2 ml of 0.01 M, 5, 5’-dithiobis 2-nitrobenzoic acid (DTNB) and 2.7 ml phosphate buffer (0.1 M, pH8.0) in a total volume of 3.0 ml. The mixture was kept at room temperature for 10 min. The yellow color that developed was measured at 412 nm. The results were expressed as micromoles of GSH per milligram of protein.

**Preparation of Post Mitochondrial Supernatant.** Livers removed aseptically from all the groups were rinsed in isotonic saline solution and weighed. A 10% (w/v) tissue homogenate in each case was prepared in 0.1 M phosphate buffer (pH7.4) using a Potter Elvehjen homogenizer. An aliquot of the liver homogenate was used for the estimation of lipid peroxidation and GSH levels. For the estimation of SOD, post mitochondrial preparation was made. For this, the remaining tissue homogenates were centrifuged at 10,500 g for 20 min at 4°C in a refrigerated centrifuge. The supernatants thus obtained were called as the post mitochondrial supernatants (PMS).

**Measurement of superoxide dismutase (SOD).** SOD activity was assayed according to the method of Kono (21). The reaction was initiated by the addition of 0.5 ml of hydroxylamine hydrochloride to the reaction mixture containing 2.0 ml nitroblue tetrazolium (NBT) and 0.1 ml PMS of liver homogenate. Change in absorbance was measured spectrophotometrically at 560 nm. SOD activity was expressed as units of SOD per milligram of protein where one unit of activity is defined as the amount of SOD required to inhibit the rate of reduction of NBT by 50%.

**Measurement of catalase activity.** The catalase activity was assayed by the method of Luck (22). The assay mixture consisted of 3.0 ml H_2_O_2_-phosphate buffer (0.05 M, pH7.0) and 0.05 ml of PMS taken directly in a cuvette. Change in absorbance was recorded spectrophotometrically at 240 nm. The results were expressed as millimoles of H_2_O_2_ decomposed per min per mg protein using the molar extinction coefficient of the chromophore (0.0394 mM^-1^ cm^-1^).

**Estimation of nitrite levels.** The amount of nitric oxide was determined by Griess reaction, as described by Green *et al*. (23). The assay is based on the propensity of nitric oxide to be oxidized to nitrate and nitrite under physiological conditions. For this, 400 μl aliquots of serum sample was mixed with 100 μl of distilled water and 500 μl of Griess reagent (0.1% Naphthylethylene diamine dihydrochloric acid and 1% sulphanilamide in 5% phosphoric acid) and incubated at room temperature for 10 min. Absorbance was measured at 546 nm. Nitrite levels in all the samples were quantified according to the standard graph of sodium nitrite.

**TNF-α assay.** Assay for tumor necrosis factor (TNF-α) was performed by ELISA in the liver homogenate in all the groups by commercially available cytokine assay kit (R&D Systems, USA) according to the manufacturers instructions. Briefly, standards for TNF-α were dispensed in the 96 well microtitre plates pre-coated with monoclonal antibody specific for rat TNF-α. To each of the designated wells, 50 μl of each test sample and 50 μl of assay diluent was added, the plates were sealed with acetate plate sealer and incubated at room temperature for 2 h. Plates were then washed five times with the wash buffer and 100 μl of rat TNF-α conjugate was dispensed into each well. Plates were again sealed and incubated at room temperature for 2 h, after which they were washed five times with the wash buffer and 100 μl of substrate solution was dispensed into each well. Plates were finally incubated at room temperature (in dark) for 30 min. 100 μl of the stop solution was added into each well to stop the reaction and absorbance was read at 450 nm. The results were expressed as picogram/ml of the TNF-α released. The ELISA was sensitive to 5 picogram/ml of the TNF-α released.

**Analysis of in situ cell death.** Paraffin-embedded tissue sections from all the groups were analyzed for the presence of DNA fragmentation using Terminal deoxy-transferase-mediated dUTP Nick End-Labeling (TUNEL reaction). *In situ* apoptosis detection kit (R & D, USA) was used to observe DNA fragmentation as it can differentiate between control, apoptotic and necrotic cells. Briefly, tissue samples were fixed to prevent the loss of low molecular weight DNA fragments. Cell membranes were treated with proteinase K to make the DNA accessible to the labeling enzyme. Then, biotinylated nucleotides were incorporated into the 3-OH ends of DNA fragments using streptavidin-horseradish peroxidase conjugate followed by the substrate, diaminobenzidine (DAB). DAB-stained samples were examined using a light microscope.

**Statistical analysis.** Results were expressed as mean ± SD. The inter group variation was measured by one way analysis of variance (ANOVA) followed by Fischer’s LSD test. The statistical analysis was done using Jandel Sigma Stat Statistical Software version 2.0. Statistical significance of the results were calculated atleast at *p*<0.05.

## RESULTS

LPS dosage and time of sacrifice of rats were standardized using different concentrations of LPS (i.v.). LPS at a dose of 10 mg/kg b. wt caused liver damage in 8 hr as assessed by liver damage markers viz. ALT, AST, ALP and histopathological examination. It was also observed (data not shown) that administration of 0.25% ethanol (vehicle for melatonin) i.p. to control animals did not cause any significant effect on any of the parameter studied in the present investigation when compared with animals not given any specific treatment.

### Effect of melatonin on liver damage markers

**Figure 1:**
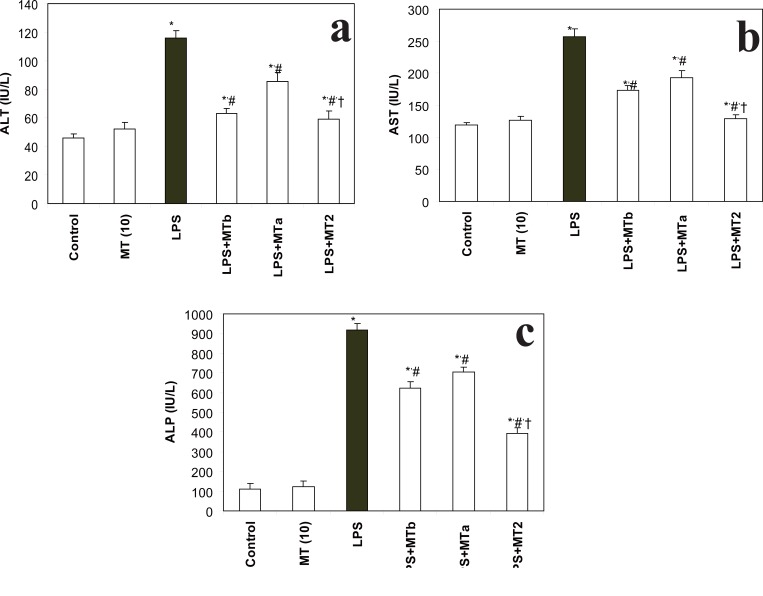
**a,** Effect of melatonin on liver ALT levels in LPS treated rats. Values are expressed as mean ± S D. *p<0.05 vs vehicle and MT *per se*, #*p*<0.05 vs LPS; † *p*<0.05 vs LPS+MTa. **b,** Effect of melatonin on liver AST levels in LPS treated rats. Values are expressed as mean ± SD. *p<0.05 vs vehicle and MT *per se*, #*p*<0.05 vs LPS; †*p*<0.05 vs LPS+MTa, LPS+MTb. **c**, Effect of melatonin on liver ALP levels in LPS treated rats. Values are expressed as mean ± SD. *p<0.05 vs vehicle and MT *per se*, #*p*<0.05 vs LPS; † *p*<0.05 vs LPS+MTa, LPS+MTb.

**AST, ALT and ALP levels.** Levels of AST, ALT and ALP were found to be significantly (*p*<0.05) raised in LPS challenged group as compared to the control group. The increase in each parameter was 2.1, 2.5 and 8.4 fold, respectively. However, melatonin significantly attenuated the LPS induced rise in these liver marker enzymes while melatonin *per se* had no effect as such on the liver enzyme levels. Maximum effect on AST, ALT and ALP levels was observed when one prophylactic and one therapeutic dose of melatonin were given to LPS challenged rats (LPS+MT2) (Fig. 1a-c). Interestingly, the decrease in the levels of hepatic markers (AST, ALT and ALP) was almost 2 fold (1.98, 2.1 and 2.2, respectively) in LPS + MT2 group when compared to the LPS group.

**Figure 2:**
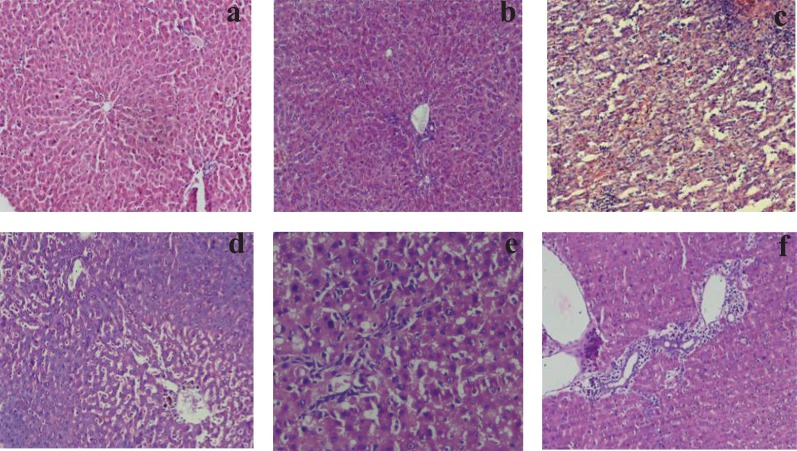
**a,** Photomicrograph of the normal / Control liver of Wistar rat showing normal liver morphology (100 ×); **b,** Photomicrograph of rat liver of melatonin *per se* group showing normal liver morphology (100 ×); **c,** Photomicrographs of liver from rat 8 hours after LPS administration, showing lymphocytic infiltration and degenerative changes i.e. necrosis in hepatocytes, Kupffer cell hyperplasia and heavily infiltrated portal tract (100 ×); **d,** Photomicrograph of liver from rat treated with melatonin 30 min before LPS challenge showing prominent central vein surrounded by excessive lymphocytes within the dilated sinusoids (100 ×); **e,** Photomicrograph of liver from rat treated with melatonin 2 hr after LPS challenge showing moderate inflammation in the portal tract and surrounding liver cell cords (100 ×); **f,** Photomicrograph of liver from rat treated with melatonin 30 min before and 2 hrs after LPS challenge showing periportal inflammation and normal liver cells (100 ×).

**Histopathological studies.** Histopathological evaluation did not reveal any morphological alterations in the vehicle or the control group (Fig. 2a) and melatonin *per se* group (Fig. 2b). In contrast, livers of LPS-administered rats showed marked morphological disruption such as Kupffer cell hyperplasia, necrosis and lymphocytic infiltration (Fig. 2c). Administration of melatonin 30 min before LPS challenge resulted in significant morphological protection/treatment against LPS induced liver damage (Fig. 2d). Similar effect was seen when melatonin was injected post LPS challenge (Fig. 2e). There was marked reduction in inflammation and hepatocyte damage in all the LPS challenged rats supplemented with two doses of melatonin in MT2 group (Fig. 2f).

### Effect of melatonin on extent of lipid peroxidation

**Figure 3:**
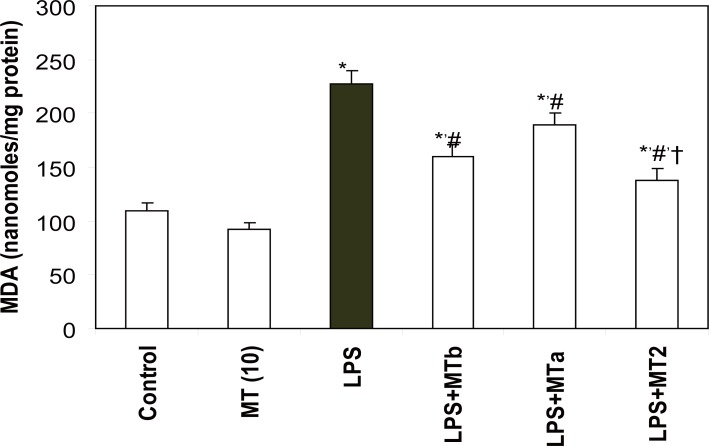
Effect of melatonin on liver MDA levels in LPS treated rats. Values are expressed as mean ± SD. ^*^p<0.05 vs vehicle and MT *per se,* #*p*<0.05 vs LPS; †*p*<0.05 vs LPS+MTa, LPS+MTb.

LPS caused a two fold increase in MDA levels as compared to control rats (from 109.0 ± 7.5 to 227.0 ± 12.0 nanomoles of MDA/mg protein). However, administration of melatonin before and after LPS challenge decreased the MDA levels by 1.7 fold as compared to the LPS group (Fig. 3).

### Effect of melatonin on the antioxidant profile

**Figure 4:**
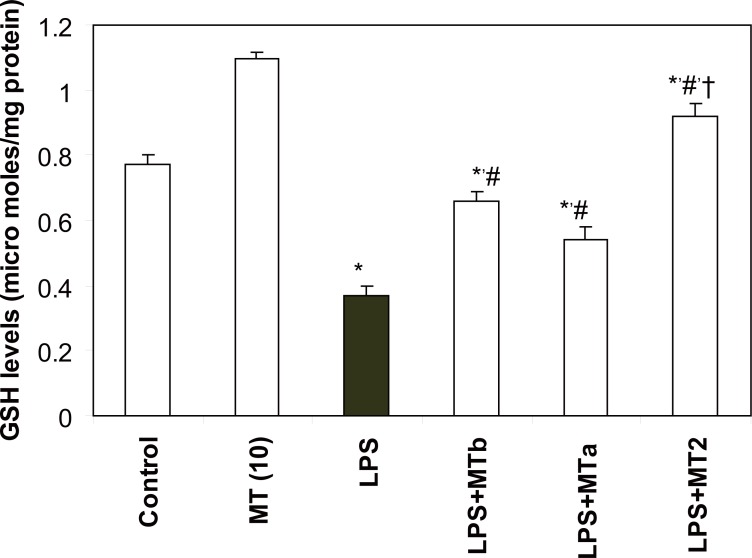
Effect of melatonin on liver GSH levels in LPS treated rats. Values are expressed as mean ± SD. ^*^p<0.05 vs vehicle and MT *per se,* #*p*<0.05 vs LPS; †*p*<0.05 vs LPS+MTa, LPS+MTb.

**GSH levels.** LPS caused a significant decrease in hepatic GSH levels as compared to the control rats (from 0.77 ± 0.03 to 0.37 ± 0.03 micromoles of GSH/mg protein). Administration of melatonin elevated levels of GSH in all the LPS challenged groups which received melatonin supplementation. However, maximum effect (2.5 fold) of melatonin was observed in the group receiving two doses of melatonin, one before and second after LPS injection (Fig. 4).

**Figure 5:**
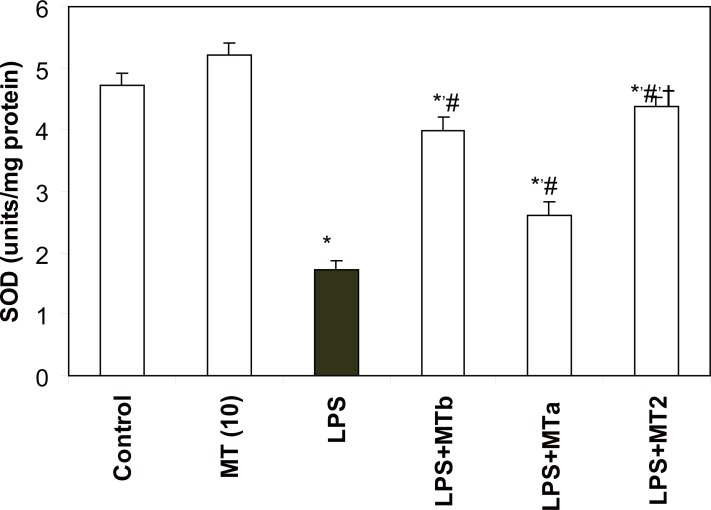
Effect of melatonin on liver SOD levels in LPS treated rats. Values are expressed as mean ± SD. ^*^p<0.05 vs vehicle and MT *per se,* #*p*<0.05 vs LPS; † *p*<0.05 vs LPS+MTa, LPS+MTb.

**Figure 6:**
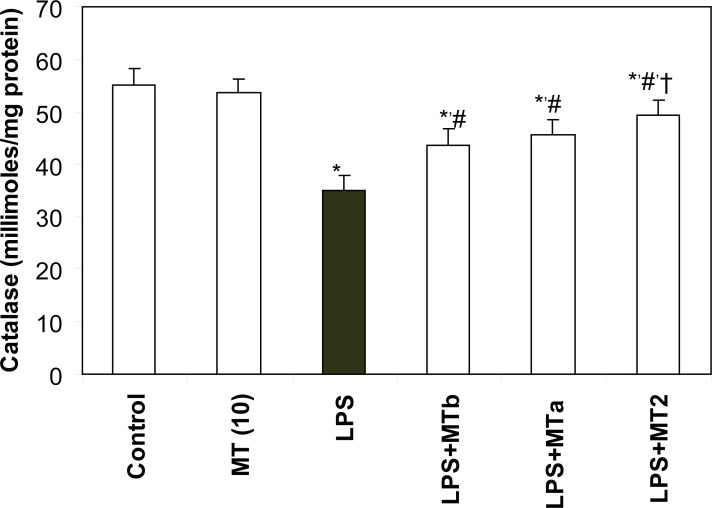
Effect of melatonin on liver catalase levels in LPS treated rats. Values are expressed as mean ± SD. ^*^p<0.05 vs vehicle and MT *per se,* #*p*<0.001 vs LPS; †*p*<0.05 vs LPS+MTb.

**SOD and catalase activities.** LPS significantly reduced the levels of liver SOD and catalase as compared to the control group (2.7 and 1.5 folds, respectively). Melatonin treatment in LPS challenged rats increased the SOD and catalase levels in both pre and post-LPS challenged groups (2.56 and 1.4 fold, respectively) (Fig. 5, 6). Again maximum effect was observed in the animals receiving two melatonin doses (before and after LPS challenge). Same trend was observed for the levels of catalase also. However, animals in the group treated with only melatonin did not show any change in the antioxidant enzyme levels.

### Effect of melatonin on nitrite levels

**Figure 7:**
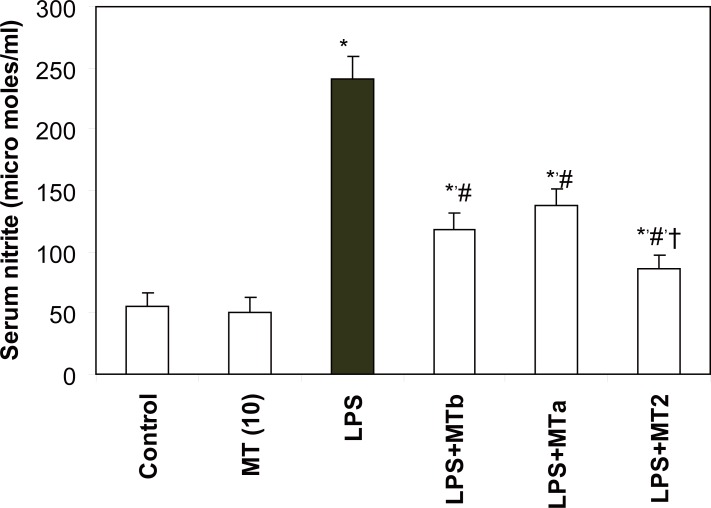
Effect of melatonin on serum nitrite levels in LPS treated rats. Values are expressed as mean ± SD. ^*^p<0.001 vs vehicle and MT *per se,* #*p*<0.001 vs LPS; † *p*<0.001 vs LPS+MTa, LPS+MTb.

Levels of serum nitrite were found to be significantly higher in LPS challenged group as compared to the control counterpart (241.2 ± 18.6 and 55.7 ± 10.4 micromoles/mg protein, respectively). Supplementation of melatonin, both pre and post- LPS challenge, markedly decreased the levels of nitrite in serum (Fig. 7).

### Effect of melatonin on LPS-induced changes in TNF-α

**Figure 8:**
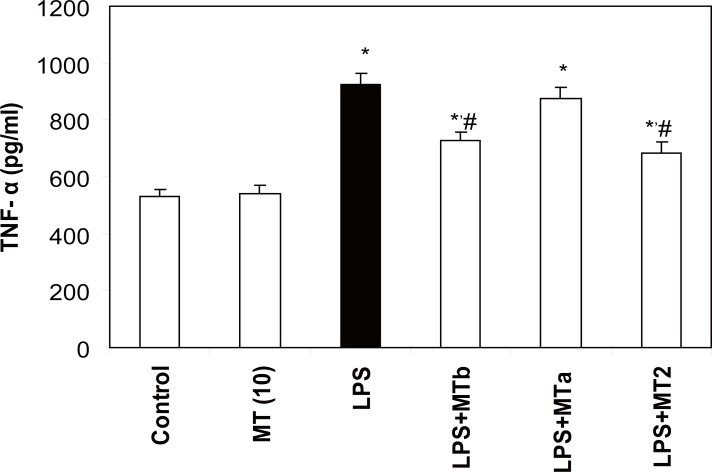
Effect of melatonin on TNF - α levels in LPS treated rats. Values are expressed as mean ± SD. ^*^p<0.05 vs vehicle and MT *per se,* #*p*<0.05 vs LPS.

LPS challenge caused a marked rise in the levels of TNF-α as compared to the control group (1.7 fold). However, the administration of melatonin both pre and post- LPS challenge (i.e. LPS Vs LPS+ MT 2 group) significantly decreased the levels of TNF-α in liver PMS by 1.1 fold (Fig. 8).

### Potential of melatonin in attenuating the cellular necrosis

**Figure 9:**
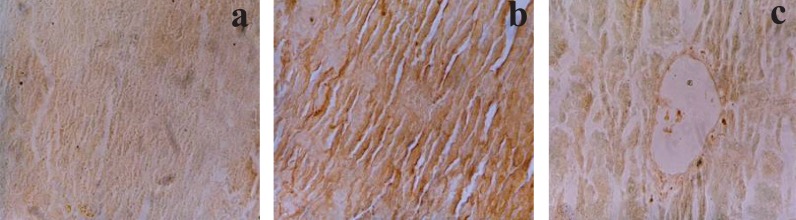
**a,** Photomicrograph of control rat liver after TUNEL reaction showing normal light brown staining (250 ×). **b,** Photomicrograph of liver 8 hrs after LPS administration to rats, showing dark brown staining depicting hepatic necrosis (250 ×). **c,** Photomicrograph of liver from rat treated with melatonin and LPS challenged showing very light to normal uptake of stain depicting considerable prevention from necrosis (250 ×).

Liver sections from the control groups showed brown stain which was homogenous throughout the tissue section, depicting normal growing cell (Fig. 9a). However, the cells treated with endotoxin showed overall very dark brown color which was spread throughout the cell/tissue depicting hepatic necrosis (Fig. 9b). In contrast, the tissues treated with melatonin showed very light to normal uptake of stain with some areas consisting of greater uptake of the brown coloration depicting a low grade necrosis (Fig. 9c).

## DISCUSSION

Considering the multifaceted free radical scavenging properties of melatonin, the present study was designed to investigate the antioxidant potential of the pineal hormone melatonin on LPS induced hepatotoxicity in terms of biochemical, morphological and immunological parameters. Administration of melatonin resulted in a marked reduction of acute liver injury, as demonstrated by significant decrease in serum levels of AST, ALT and ALP. These results are in accordance with the earlier studies where similar decrease in liver enzyme levels was reported with melatonin administered into the rat sepsis model established after cecal ligation and puncture (15). In our study, a significant reduction in the enzyme levels was detected with melatonin administration before and after the LPS challenge confirming the findings of Crespo *et al*. (13). Protective effects of melatonin in reducing the levels of hepatic marker correlated well with the histological findings in the present study. Melatonin reduced the PMN infiltration which is in accordance with results reported by Sewerynek *et al*. (12). This is important because the extent of liver damage following LPS administration is directly proportional to number of PMNs (24) as these have been shown to produce large amount of proinflammatory cytokines involved in hepatotoxicity.

Melatonin has been reported to detoxify a variety of free radicals including the hydroxyl radical, singlet oxygen, peroxynitrite anion and nitric oxide (25, 26). The study carried out by Crespo *et al*. (13) showed decrease in LPO by melatonin in lungs only, while showing no effect in case of liver. However in the present study, melatonin has been observed to significantly decrease the LPS induced increase in liver MDA levels in both pre and post LPS challenged and melatonin administered groups. This may be due to the property of melatonin to scavenge HO^•^ and to protect DNA from oxidative damage (27). Similar effect of melatonin has been studied by Sener *et al*. (15), who reported the decrease in MDA levels in septic rat models treated with melatonin.

Melatonin has also been found to stimulate the activities of cell oxidative enzymes i.e. SOD, catalase, and GSH-Px and increase gene expression that improves the total antioxidant capacity of the organism (9, 28, 29, 30). GSH, the most important cellular antioxidant, functions either by protecting cells from lipid peroxidation or by protecting protein sulphydryl group from being oxidized by these radicals (31). In the present study, it was observed that melatonin restored the levels of GSH in LPS administered rats. This observation is in accordance with the one reported by Sewerynek *et al*. (12). These workers had also shown an increase in GSH levels by melatonin supplementation after LPS injection in phenylbarbituric acid treated rats. Significant decrease in the activities of SOD and catalase was also observed in our study. This may result in hampered dismutation of superoxide anions and inefficient detoxification of H_2_O_2_ which results in formation of OH^•^ ions enhancing the peroxidation of membrane lipids thereby leading to oxidative damage in many tissues (7). However, melatonin was observed to significantly increase the levels of SOD and catalase in both pre and post LPS challenge in the present investigation. The increase was more significant in the levels of SOD in comparison to the levels of catalase. Thus, the role of SOD seems to be more pronounced in combating the oxidative stress in our study.

Nitric Oxide (NO) is an important messenger regulating nervous, immune, and cardiovascular homeostasis (32). LPS causes induction of iNOS in Kupffer cells and hepatocytes (13, 33, 34) which results in over production of NO that has been implicated in the pathogenesis of circulatory shock. However, melatonin has been observed to inhibit expression of iNOS in liver and thus decreasing the nitrite levels in endotoxemic rat models (13). The present study also showed that melatonin significantly inhibited LPS induced increased serum nitrite levels.

Early phase response to LPS is mediated by cytokines such as TNF-α, a multifunctional molecule secreted by activated macrophages, monocytes, neutrophils and NK-cells. In addition to its direct cytotoxic effects, it is able to induce chemokines, macrophage chemotactic protein-1 and vascular cell adhesion molecule-1, which are the key to hyper inflammation and consequent liver damage (9). Cellular sensitivity or resistance to TNF-α is correlated with decreased or increased levels of SOD respectively (35, 36). In the present study also, increased levels of TNF-α after LPS injection correlated with the increased level of peroxidation and decreased activities of SOD and catalase. Melatonin significantly decreased the levels of TNF-α in both pre and post LPS challenge groups. Following melatonin supplementation, decreased TNF-α level along with decreased NO levels might have resulted into decreased LPO.

Evidence that TNF-α induces cell death is provided by the studies of Song *et al*. (37), who reported TNF-α generation along with parallel increase in iNOS mRNA expression and nitric oxide production induced cell death in cardiomyocytes of wild type mice. Suchuerwegh *et al*. (38) have also suggested a significant role of NO in cytokine (TNF-α and IL-1) induced bovine chondrocytes apoptosis. In a recent study (17), melatonin has been shown to attenuate GalN/LPS- induced hepatic apoptosis, measured by inhibition of caspase-3 activities and attenuation of DNA laddering. In contrast to their findings, in the present study, TNF-α mediated necrotic cell death was observed in the LPS administered rats as evidenced by TUNEL assay. Following melatonin administration, very low grade necrosis was depicted in the liver cells from rats. This may be attributed to the ability of melatonin to protect polyunsaturated fatty acids in cellular membranes along with nuclear and mitochondrial DNA from free radical damage by directly sequestering the toxic radicals (18, 30).

The results of the present study indicate that the hepatic necrosis caused by LPS may be due to activation of liver cells generating higher levels of TNF-α resulting in accumulation of free radicals, along with a decrease in levels of antioxidants culminating into oxidative stress. The oxidative damage thus induced, was modulated by supplementation of melatonin, indicating its protective potential. Thus, our findings suggest that melatonin may effectively protect against LPS toxicity in terms of biochemical, morphological and immunological damage and therefore, may have clinical application in a variety of diseased conditions where cellular damage is a consequence of free radicals.
